# 

**Published:** 1871-06

**Authors:** 


### ARTICLE III.

### *The White Sulphur Springs Meeting.*

Dr. W. H. Morgan, President of the American Dental Association, requests us to give some information concerning the best routes for reaching the "White Sulphur," where the 11th annual meeting will be held, commencing on the 1st day of August next, at 10 o'clock A. M.

These springs are situated in Greenbrier County, Virginia, at the present terminus of the Chesapeake and Ohio railroad, the nearest town of any size being Staunton, in Augusta County, through which town it is necessary to pass when going by rail. Delegates from the East, West and North must proceed to Washington city and take the Orange, and Alexandria railroad as far as Gordonsville, where they meet with the Chesapeake and Ohio railroad which will carry them to the springs.

Delegates from the South proceed to Richmond Va., Lynchburg Va., or Knoxville Tenn. Those who go by Richmond, will proceed on the Virginia Central railroad to Gordonsville, and thence by the Chesapeake and Ohio road to the springs. Those who go by Lynchburg will take the Orange and Alexandria railroad to Charlottsville, and then the Chesapeake and Ohio road to the springs. Those who go by way of Knoxville will take the Virginia and Tennessee railroad to Lynchburg Va., and the Orange and Alexandria railroad to Charlottesville, where they meet the Chesapeake and Ohio road.

By the Knoxville route, delegates will also find at the "Montgomery White" on the Virginia and Tennessee railroad, in Montgomery County Va., a daily line of stages which will carry them direct to the "White Sulphur," a distance of sixty five miles, saving considerable travel by the railroad to Charlottesville.

---

### ARTICLE IV.

### *Kansas State Dental Society.*

By Dr. J. D. Patterson, Rec. Secretary.

*Editor of Amer. Jour. of Dental Science:*—The dentists of this State met in convention, at Lawrence, on the 2nd of May last, and organized a State Dental Society. Den tists present, Drs. H. H. Marvin and A. M. Callahan, of Topeka; Dr. J. H. Sawyer, of Atchison; Dr. E. C. Fuller, of Fort Scott, and Drs. J. B. Wheeler, and J. D. Patterson, of Lawrence. A permanent organization was effected, and named, *The Kansas State Dental Society.* The Code of Ethics adopted was that of the American Dental Association, and the Constitution and By-laws similar to that in other standard local societies. Dr. J. B. Wheeler was elected President; Dr. W. H. Marvin, Vice-President; Dr. J. H. Sawyer, Treas.; J. D. Patterson, Recording Sec'y.; Dr. E. C. Fuller, Corresponding Sec'y. Dr. Fuller was elected delegate to the American Convention, for 1871. A vote of thanks was tendered Dr. Marion for inaugurating the movement for organization. The first semi-annual meeting will be held at Topeka, commencing on Sept. 12th, 1871; annual meeting at Atchison on May 2nd, 1872. After all necessary business was transacted, an irregular discussion was held, with an exchange of opinions on dental subjects. Members were highly pleased with the success of this movement, placing the profession in the State of Kansas on a footing with that in older States, by thus organizing on *a solid basis* a State Society. Cordial invitation for co-operation by the profession is extended by the Society.

The Secretary was instructed to furnish reports of this meeting to the leading dental journals of the country.

---

### ARTICLE V.

### *South Carolina State Dental Association.*

At the anniversary meeting of the South Carolina State Dental Association, held in this city on the 10th, 11th, and 12th April, the following gentlemen were elected to serve for the ensuing year:

*President*, Wm. C. Wardlaw, Abbeville; 1*st Vice-President*, Thomas T. Moore, Columbia; 2*nd Vice-President*, B. A. Muckenfuss, Charleston; *Corresponding Sec'y*, Theodore F. Chuypein, Charleston; *Recording Sec'y*, O. J. Bond, Marion; *Treasurer*, W. J. Beann, Charleston.

THEODORE F. CHUYPEIN, Corresponding Sec'y,
South Carolina State Dental Association.

### ARTICLE VI.

### *Boston Dental College.*

The Commencement of this college was celebrated on the ninth day of March last, by dissertations of the graduating Class and addresses of professors and friends.

By reason of some circumstances which it was not possible to control, the graduating class was not so large as usual. The dissertations, however, of those who did graduate, evinced a thorough knowledge of the profession and preparation for its practice.

Professors W. H. Atkinson and Wetherbee, delivered excellent addresses. A very interesting feature of the occasion, was the presentation by the College, of a very valuable and costly gold watch, to the President, D. S. Dickerman, D. D. S., M.D., which service was performed most eloquently and appropriately, by George B. Harriman, D.D.S., M. D., who has been elected to the new Professorship of Microscopical Anatomy.

The following gentlemen received the degree of Doctor of Dental Surgery: Darius F. Drake, Boston, Mass.; John Q. Dickerman, Taunton, Mass.; Charles N. Pierce, Portland, Me.; Alonzo H. Sylvester, Boston, Mass.; David G. Williams, Boston, Mass.; Fred. A. Locke, Portland, Me. H.

---

### ARTICLE VII.

### *American Dental Convention.*

The seventeeth annual meeting of the American Dental Convention will be held at Saratoga, N. Y. commencing on Wednesday the 9th day of August 1871.

All dental practitioners are entitled to the priveleges of the Convention, and invited to present any matters of general professional interest.

